# Effect of Epidural Analgesia on the Duration of Induced Labor in Primiparous and Multiparous Women: A Retrospective Study

**DOI:** 10.7759/cureus.93351

**Published:** 2025-09-27

**Authors:** Ayumi Nakago, Tomohiro Chaki, Toshiya Kawagishi, Hiroshi Nagai, Kenjiro Nakago, Yuko Nawa

**Affiliations:** 1 Anesthesiology, Ebetsu City Hospital, Ebetsu, JPN; 2 Anesthesiology, Sapporo Medical University School of Medicine, Sapporo, JPN; 3 Obstetrics and Gynecology, Ebetsu City Hospital, Ebetsu, JPN; 4 Anesthesiology, Hokkaido Medical Center for Children Health and Rehabilitation, Sapporo, JPN

**Keywords:** delivery outcomes, epidural analgesia, induced labor, labor duration, primiparous and multiparous women, retrospective study, second stage of labor

## Abstract

Context

Epidural analgesia is commonly used during labor; however, its effect on the duration of labor, particularly in induced labor, remains unclear. Most previous studies have focused on spontaneous labor, with limited data on induced labor. Notably, very few studies have directly investigated how epidural analgesia affects labor duration and delivery outcomes in the setting of planned labor induction.

Aims

This study aimed to elucidate the effects of epidural analgesia on labor duration and delivery outcomes in primiparous and multiparous women undergoing labor induction.

Settings and design

A retrospective study was conducted at our hospital, including singleton pregnancies in which labor induction was performed between December 2020 and November 2021.

Methods and materials

Oxytocin and, if needed, oral prostaglandin E2 were used for labor induction. Epidural analgesia was administered using both single-shot and continuous infusion techniques. The primary outcome was the second stage of labor. Secondary outcomes included the duration of the first stage, rates of instrumental delivery, Kristeller maneuver, cesarean section, and Apgar scores at one and five minutes.

Statistical analysis

Continuous variables were analyzed using unpaired t-tests, and categorical data using chi-square tests. Propensity score matching was applied to adjust for confounding factors.

Results

Among 243 cases, epidural analgesia significantly prolonged the second stage of labor by 19 minutes in multiparous women (p=0.002), but had no effect in primiparous women (p=0.414). No significant differences were observed in any secondary outcomes. Results were consistent after propensity score matching.

Conclusion

The second stage of labor was not prolonged in primiparous women, while a modest prolongation occurred in multiparous women. However, there were no significant differences in other delivery-related outcomes, including rates of instrumental delivery or cesarean section.

## Introduction

The use of labor analgesia, which provides pain relief for women during labor, is increasing worldwide. In the United States, the rate of labor analgesia usage has reached 73.1% [1], and the rate is even higher in France at 83.8% [2]. In contrast, the rate of labor analgesia in Japan was only 6.1% in 2016, which is relatively low, but it has been steadily increasing [3]. This upward trend indicates a growing recognition of the benefits of labor analgesia, such as improved maternal satisfaction and reduced labor-related stress, highlighting the importance of investigating the impact of labor analgesia on labor outcomes.

Previous research consistently suggests that labor analgesia, particularly epidural analgesia, extends the second stage of labor [4]. However, most previous studies focused on the effects of labor analgesia during spontaneous labor.

Indeed, early studies on induced labor have suggested a significant prolongation of labor duration [5], prolonging the second stage of labor, accompanied by a decrease in normal vaginal delivery rates and a marked increase in forceps deliveries [6]. However, it is important to note that these findings are derived from older data.

Retrospective studies show that epidural analgesia in induced labor is associated with longer labor stages and higher rates of instrumental and cesarean delivery [7]. Nonetheless, these findings are complex, as they are based on data where significant baseline differences in maternal age and BMI persisted even after attempts at statistical adjustment. It is also possible that these differences, rather than the epidural analgesia itself, could be the actual cause of the outcomes, leaving gaps in the understanding of the impact of epidural analgesia on induced labor. Induced labor, which involves the administration of uterotonics, provides a unique context for eliminating certain confounding factors such as the unpredictability of spontaneous labor onset [8]. Recent studies have shown that induced labor may improve maternal and neonatal outcomes and reduce cesarean section rates [9]. ­

Given the anticipated increase in induced labor, particularly in Western countries [10,11], it is becoming increasingly important to clarify how epidural analgesia affects induced labor. Induced labor is often performed to address maternal or fetal conditions, and its frequency of use may further increase due to the rising rates of advanced maternal age and comorbid conditions such as gestational diabetes and hypertension. However, it has been reported that elective labor induction is associated with more pain, intrapartum interventions, caesarean delivery, and prolonged maternal stay, as compared to spontaneous labor, necessitating the use of labor analgesia [12]. Along with the expanding use of labor analgesia, further research is needed to elucidate how epidural analgesia during induced labor affects labor outcomes compared to its effects during spontaneous labor.

Therefore, the aim of this study was to determine the effects of epidural analgesia during induced labor in both primiparous and multiparous women. The hypothesis of this study is that epidural analgesia prolongs the second stage of labor in both primiparas and multiparas undergoing induced labor.

This study was previously presented as an abstract at the Annual Meeting of the Japanese Society for Perinatal Anesthesia on March 25, 2023, in Fukuoka, Japan.

## Materials and methods

This study was approved by the ethics committee of our hospital (date of approval: December 20, 2023).

This retrospective observational study included pregnant women who underwent induction of labor at Ebetsu Municipal Hospital between December 2020 and November 2021. The inclusion criteria were single pregnancy, an American Society of Anesthesiologists Physical Status (ASA PS) Classification of I or II [13,14], and a gestational age between 37 and 40 weeks. We excluded cases with severe complications such as uterine rupture or atonic bleeding, as well as multiple pregnancies. Additionally, cases in which a cesarean section was performed were excluded because an accurate measurement of labor time was not possible. Cases with missing data essential for analysis were also excluded (e.g., two cases in the primipara group were excluded due to missing data).

**Figure 1:**
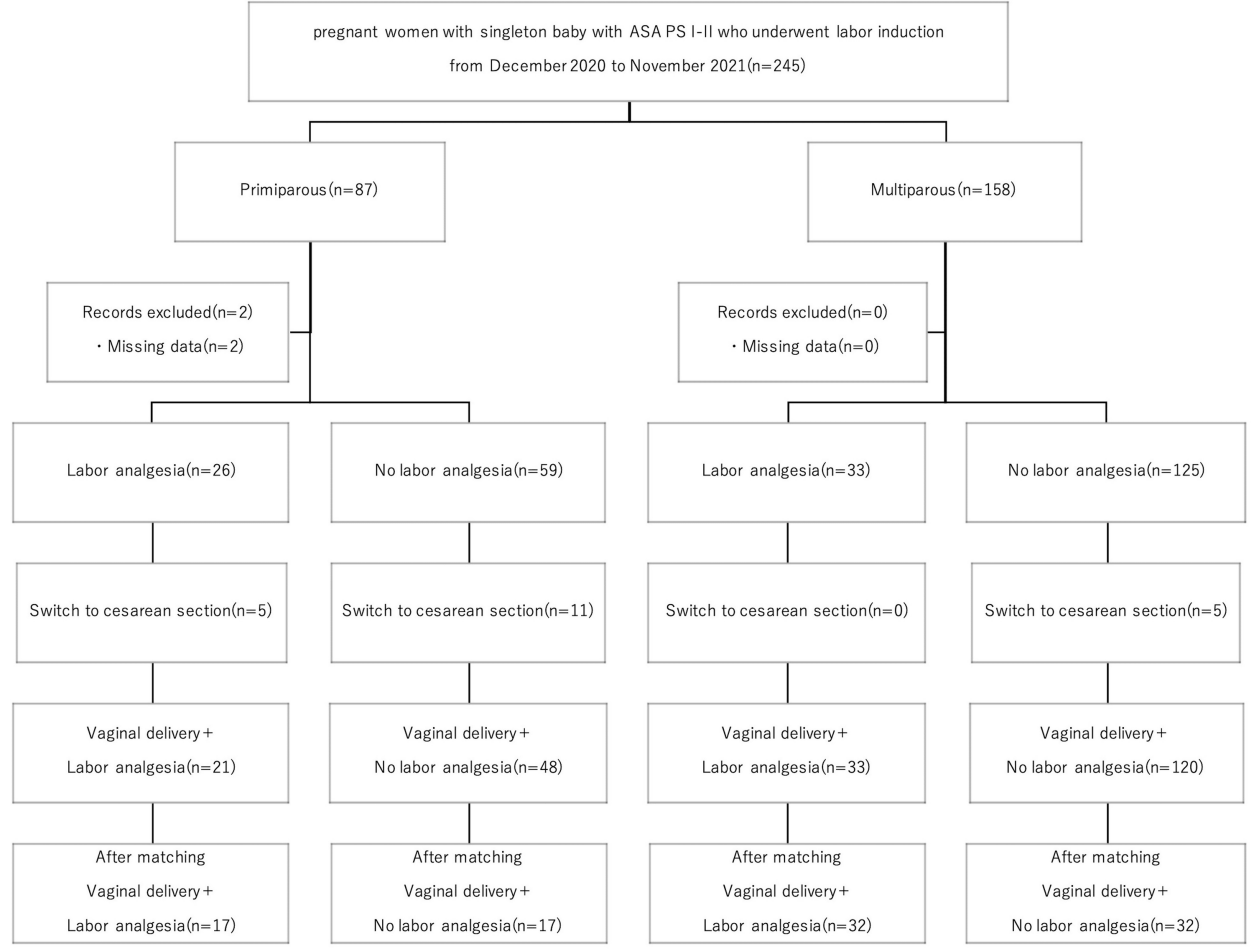
Study flow diagram of pregnant women undergoing labor induction

During the total observation period, 245 cases of induction of labor were recorded, of which 87 were primiparas and 158 were multiparas. After excluding cases based on the above criteria, 85 primiparous and 158 multiparous cases were analyzed primarily for data quality. The analysis of the duration of delivery finally included a cohort of 69 primiparous and 153 multiparous cases, as shown in Figure 1.

Methods of delivery

The pregnant women were admitted for labor induction scheduled between 37 and 40 weeks of gestation. Oral prostaglandin E2 was administered only in cases where cervical ripening was insufficient. Labor induction was initiated by inserting a metreurynter into the cervix to facilitate dilation, and oxytocin was administered in all cases to accelerate labor. Oxytocin was continuously administered until delivery. The oxytocin infusion was started at a rate of 50 mU/h, and the infusion rate was increased by 50 mU/h every 30 minutes until effective labor contractions were established, with a maximum rate of 600 mU/h. Once effective labor contractions were achieved, no further increases in the medication were made, and the labor was monitored. Labor was defined as regular uterine contractions occurring every 10 minutes. When cervical ripening and labor induction were successful, artificial rupture of membranes was frequently performed to enhance reliable labor progression. Fetal heart rate was continuously monitored in all cases.

Methods of analgesia (management of labor analgesia)

An epidural catheter (ICU Medical Japan, Tokyo, Japan) was inserted at the L3/4 interspace on the morning of admission, before the onset of labor. In cases where the dural puncture epidural (DPE) technique was applied, it was performed at the same level. The catheter was threaded 4-6 cm into the epidural space.

A single dose of analgesia was administered via an epidural catheter once the pregnant woman experienced unbearable labor pain. This single dose was not a standalone intervention but was followed by continuous epidural analgesia. One hour after the initial single dose, continuous epidural analgesia was initiated. Thereafter, additional single doses were administered each time the woman experienced unbearable labor pain (Numeric Rating Scale (NRS) pain score of 3 or higher).

The single-dose analgesia consisted of 6-8 mL of 0.2% ropivacaine (final concentration: 0.055-0.08%), 6 mL of 1% lidocaine (final concentration: 0.27-0.3%), 4 mL of fentanyl (final concentration: 9-10 μg/mL), and 2-6 mL of saline, with a total volume of 20-22 mL. Lidocaine was included in the mixture because it provides a faster onset of analgesia than ropivacaine; this strategy allowed lidocaine to cover the early phase of labor pain relief while the concomitantly administered ropivacaine took effect later. To prevent local anesthetic toxicity, the drug solution was divided into three to four doses of 5-6 mL each and was repeatedly administered as single doses until effective analgesia was achieved, defined as a NRS pain score of 2 or less.

For continuous epidural administration, a total of 300 mL of solution was used, consisting of 100-120 mL of 0.2% ropivacaine (final concentration: 0.067-0.08%), 10 mL of fentanyl (final concentration: 1.6 μg/mL), and 170-190 mL of saline. The infusion rate for continuous administration was set at 10 mL/h. When the DPE technique was applied, the concentration of ropivacaine was not fixed at a single value but was gradually adjusted, and during this period, the concentration was lowered stepwise to 0.055%.

Patient-controlled analgesia (PCA) was set at 5 mL per dose, with a lockout interval of 15 minutes. The drugs and administration methods for epidural anesthesia are shown in Table 1.

**Table 1:** Medications and administration method for epidural anesthesia These protocols represent the standard practice for epidural analgesia at our institution during the study period. NRS, Numeric Rating Scale; PCA, patient-controlled analgesia

One-shot administration	Continuous administration
Ropivacaine: 0.055-0.08%, Fentanyl: 9-10 μg/mL, Lidocaine: 0.27-0.3%. Dosage: Each 5-6 mL. Frequency: Administered every 5 minutes for 3-4 times. Additional administration: The above drugs were added 1-4 times according to the NRS pain score.	Ropivacaine: 0.067-0.08%, Fentanyl: 1.6 μg/mL. Dosage: Continuous infusion at a rate of 10 mL/hour. PCA: Continuous/intermittent administration of drugs was used. Dosage: 5 mL per administration. Lockout time: Set to 15 min.

Outcomes

The primary outcome was the duration of the second stage of labor. Secondary outcomes included the duration of the first stage of labor, rates of instrumental delivery, transition to cesarean section, and Apgar scores at one and five minutes.

Statistical analysis

The sample size was calculated using G*power 3.1 (Heinrich-Heine-University, Düsseldorf, Germany). Based on previous research, a mean difference of 16.6 minutes for the second stage of labor with a standard deviation of 22 minutes was considered clinically significant [15]. Because the application rate of labor analgesia in our institution was 25%, the allocation rate was set as 0.25. A total of 140 cases were necessary based on 1-β=0.90 and two-tailed α=0.025. Furthermore, considering a 10% dropout rate for data deviation, the required number of cases for multiparous women was calculated to be 154 cases. To ensure this required case number, the observational period was set from December 2020 to November 2021.

Continuous variables are expressed as means ± standard deviation. Categorical data are presented as absolute numbers (proportions). The first and second stages of labor durations and Apgar scores were analyzed by the unpaired t-test. The instrumental delivery rate, use of Kristeller's maneuver, and cesarean section rate were analyzed using the chi-square test. Additionally, propensity score matching based on factors including age, pre-pregnancy body mass index (BMI), and BMI at delivery was performed for primiparous and multiparous women separately. After matching, unpaired t-tests were conducted for the first and second stages of labor durations. Since unpaired t-tests were conducted separately for primiparous and multiparous groups, a significance level of two-tailed α=0.025 using Bonferroni correction was adopted for primary outcome analysis. For the secondary outcomes, a significance level of two-tailed α=0.05 was set. Statistical analysis was performed using GraphPad Prism 10 (GraphPad Software, San Diego, CA).

This study received no specific grant from any funding agency in the public, commercial, or not-for-profit sectors.

## Results

During the observational period, 245 cases of labor induction were recorded, comprising 87 primiparous and 158 multiparous cases. 

Primiparous group

Initially, 87 primiparous cases were recorded. Two cases were excluded due to data loss, resulting in 85 primiparous cases for initial analysis. Labor analgesia was administered in 26 cases (31%) among these primiparous women. The remaining 59 cases initially did not receive labor analgesia (Figure 1).

For the purpose of accurate labor duration analysis, cases that underwent cesarean section were excluded. Consequently, a total of 69 primiparous cases, including 21 labor analgesia cases and 48 non-labor analgesia cases, were finally analyzed for labor duration(Figure 1).

Multiparous group

A total of 158 multiparous cases were recorded. Labor analgesia was administered in 33 cases (21%) among these multiparous women. The remaining 125 cases initially did not receive labor analgesia (Figure 1).

Similarly, for the purpose of accurate labor duration analysis, cases that underwent cesarean section were excluded. Consequently, a total of 153 multiparous cases, including 33 labor analgesia cases and 120 non-labor analgesia cases, were finally analyzed for labor duration (Figure 1).

There was a significant difference in the ages of primiparous and multiparous women, but there was no significant difference between primiparous and multiparous women in pre-pregnancy BMI, BMI at delivery, or hospital stay to delivery duration (Table 2).

**Table 2:** Patient background The data are presented as mean±standard deviation. BMI, body mass index

Characteristics	Non-labor analgesia	Labor analgesia
Primiparous	n=48	n=21
Age (years)	28.9±5.0	31.5±4.9
Pre-pregnancy BMI (kg/m^2^)	21.5±2.9	21.5±3.7
BMI at delivery (kg/m^2^)	25.9±3.0	25.4±3.6
Hospitalization to delivery days	2.4±1.1	2.7±1.7
Multiparous	n=120	n=33
Age (years)	32.4±4.6	32.7±3.6
Pre-pregnancy BMI (kg/m^2^)	22.2±3.2	21.0±3.3
BMI at delivery (kg/m^2^)	26.0±3.3	26.1±3.6
Hospitalization to delivery days	1.7±1.1	1.5±0.5

In the second stage of labor, no significant difference was found in the primiparous women (non-labor analgesia: 69±57 min vs. labor analgesia: 81±59 min; mean difference: 12 min; 95% CI: -18-42 min; p=0.414). However, in the multiparous women, there was a significant extension in cases in which labor analgesia was administered (non-labor analgesia: 15±31 min vs. labor analgesia: 34±30 min, mean difference: 19 min; 95% CI: 7-31 min; p=0.002) (Table 3). Additionally, the duration of the first stage of labor, the rate of assisted vaginal delivery, the use of Kristeller's maneuver, the cesarean section rate, and the Apgar scores showed no significant differences between cases with and cases without labor analgesia, both in primiparous and multiparous women (Table 3).

**Table 3:** The impact of analgesia on labor progress in primiparous and multiparous women Statistical analysis was performed using unpaired t-tests for continuous variables and Fisher's exact tests for categorical variables. The data are presented as mean±standard deviation and absolute values (%) with CI.

Outcomes	Non-labor analgesia	Labor analgesia	Mean difference	Odds ratio	95% CI	P-value
Primiparous (n=69)	n=48	n=21				
First stage of labor (min)	377±301	349±323	-28	-	-189 to 133	0.727
Second stage of labor (min)	69±57	81±59	12	-	-18 to 42	0.414
Assisted vaginal delivery	10 (17%)	6 (23%)	-	1.47	0.44 to 4.58	0.553
Kristeller’s maneuver	14 (24%)	7 (27%)	-	1.18	0.45 to 3.55	0.789
Apgar score 1 min	8.2±0.6	8.3±0.7	0.1	-	-0.2 to 0.4	0.495
Apgar score 5 min	9.0±0.4	9.0±0.5	0.0	-	-0.2 to 0.2	0.952
Multiparous (n=153)	n=120	n=33				
First stage of labor (min)	226±130	248±175	-21	-	-76 to 33	0.439
Second stage of labor (min)	15±31	34±30	19	-	7 to 31	0.002
Assisted vaginal delivery	0 (0%)	1 (3%)	-	-	-	0.210
Kristeller’s maneuver	3 (2%)	1 (3%)	-	1.26	0.09 to 8.68	>0.999
Apgar score 1 min	8.6±0.5	8.5±0.6	-0.1	-	-0.3 to 0.1	0.392
Apgar score 5 min	9.1±0.3	9.1±0.3	0.0	-	-0.1 to 0.1	0.869

Cases converted to cesarean section were not included in the "n" values presented in Table 3. Therefore, while the cesarean section conversion rates are not explicitly detailed within Table 3, they are described below.

In primiparas, 11 of 59 (19%) undergoing labor without analgesia and 5 of 26 (19%) undergoing labor with analgesia converted to cesarean section. For this group, the odds ratio was 1.04 (95% CI 0.36 to 3.29), and the P-value was >0.999.

In multiparas, 5 of 120 (4%) undergoing labor without analgesia and 0 of 33 (0%) undergoing labor with analgesia converted to cesarean section. For this group, the odds ratio and its 95% CI could not be calculated, and the P-value was >0.580.

No significant difference in the conversion rate to cesarean section was observed within either the primiparous or multiparous groups.

After propensity score matching, no significant differences were found in the first and second stages of labor for primiparous cases. In multiparous cases, there was no significant difference in the first stage of labor, but the second stage of labor was significantly longer in cases with epidural analgesia (with epidural analgesia: 34±30 min vs. without epidural analgesia: 13±9 min, mean difference: 21 min; 95% CI: 10-30 min; p<0.001) (Table 4).

**Table 4:** The impact of analgesia on labor progress after propensity score matching The data are presented as mean±standard deviation. Statistical analysis was performed using unpaired t-tests for continuous variables.

Outcomes	Non-labor analgesia	Labor analgesia	Mean difference	95% CI	P-value
Primiparous (n=34)	(n=17)	(n=17)	-		
First stage of labor (min)	377±304	362±349	-15	-214 to 243	0.896
Second stage of labor (min)	68±52	85±62	17	-23 to 57	0.395
Multiparous (n=64)	(n=32)	(n=32)	-		
First stage of labor (min)	237±133	253±175	17	-61 to 95	0.665
Second stage of labor (min)	13±9	34±30	21	10 to 33	<0.001

## Discussion

This study showed that there was no prolongation of either the first stage or second stage of labor in primiparous women who received epidural analgesia during induced labor. This study also showed that while the first stage was not prolonged, the second stage was extended by an average of 19 minutes in multiparous women. Additionally, in both primiparous and multiparous women undergoing induced labor, epidural analgesia did not increase the rates of instrumental delivery, Kristeller maneuver, or transition to cesarean section or change the Apgar score.

Amin-Somuah et al. reported that the labor analgesia group had a 15-minute longer second stage than that in the parallel opioid-only group [4]. Halpern et al. conducted a meta-analysis of studies and showed that neuraxial analgesia is associated with a longer (~16 minutes) second stage of labor than that with parenteral opioids [16]. Our study, in contrast to those findings, showed no significant extension in the second stage of labor duration for primiparous women receiving labor analgesia. Similarly, Elliott et al. reported that epidural analgesia extended the first stage of labor by 30 minutes and extended the second stage by 15 minutes [8]. However, our study showed no extension in the duration of the first stage of labor for either primiparous or multiparous women and no significant difference in the duration of the second stage of labor for primiparous women. These discrepancies may be attributed to two key factors: first, the effects of labor induction; and second, the use of lower concentrations of analgesic medication in our study. In the context of labor induction, uterotonic agents are administered from the onset of labor, which enables prompt and proactive augmentation of uterine contractions as needed. This may have helped mitigate the uterine inertia sometimes caused by neuraxial analgesia, thereby reducing its impact on labor progression. The use of lower concentrations of local anesthetic may have reduced motor block and preserved maternal expulsive efforts, which could partly explain the absence of significant prolongation of the second stage of labor in primiparous women in this study. The use of lower concentrations of local anesthetic may have reduced motor block and preserved maternal expulsive efforts, which could partly explain the absence of significant prolongation of the second stage of labor in primiparous women in this study. In our institution, the transition from conventional epidural analgesia to the DPE technique was an important step that enabled the safe use of such lower concentrations. While conventional epidural analgesia provided adequate pain relief, it was sometimes less effective in the sacral region, particularly during the second stage of labor. After transitioning to the DPE technique in August 2021, sacral coverage improved, and pain control became more consistent. This allowed us to safely titrate the concentration of ropivacaine stepwise from 0.08% to 0.055% during the study period while maintaining effective analgesia and minimizing the impact on uterine contractions and maternal expulsive efforts (Table 1). Previous studies using the DPE technique have generally reported effective labor analgesia with ropivacaine concentrations of 0.075-0.125% [17,18]. This discrepancy may be explained by several factors, including the transition from conventional epidural analgesia to the DPE technique, the combination of single-dose boluses with continuous infusion, and the admixture of lidocaine, which provided faster onset and facilitated effective analgesia when combined with ropivacaine [19-21]. Although effective, this combination requires caution, as possible interactions have been reported [22].

Successful labor depends on three factors: maternal expulsive efforts (power), fetal characteristics (passenger), and pelvic anatomy (passage) [23]. Neuraxial analgesia can negatively affect power and passage, potentially leading to uterine inertia or reduced pushing force. From an anesthesiologist’s perspective, minimizing total anesthetic consumption remains essential to reduce its impact on labor progression.

The results of this study showed that epidural analgesia during labor induction was associated with an average 19-minute extension of the second stage in multiparous women, who typically have shorter labor durations. However, there was no significant prolongation in primiparous women, who generally experience longer labor.

According to the guidelines of the American College of Obstetricians and Gynecologists and the Society for Maternal-Fetal Medicine, a second stage of up to two to three hours is considered normal in multiparas receiving neuraxial analgesia [24]. Given that spontaneous second-stage durations typically range from 20 to 60 minutes, a 19-minute extension still results in a total duration well below the two-hour threshold.

Therefore, this degree of prolongation is unlikely to be clinically meaningful, and epidural analgesia can be safely offered during labor induction without undue concern about labor delay.

However, several limitations might have affected the results of our study. First, being a retrospective study, unadjusted background factors might exist despite propensity score matching. Second, the management methods for intrapartum analgesia were not standardized throughout our study's observational period. While comparisons between different labor analgesia methods were warranted, inadequate sample size hindered us from conducting this analysis. Future randomized comparative trials considering these aspects are necessary.

## Conclusions

This study suggests that epidural analgesia during planned labor induction has minimal impact on labor duration when using a low-concentration local anesthetic protocol. Multiparous women showed only a modest extension of the second stage, which remained within clinically acceptable limits, and no prolongation was observed in primiparous women. Other delivery outcomes, including instrumental delivery, cesarean section, and neonatal Apgar scores, were unaffected. The transition to the DPE method during the study period may have facilitated the adoption of a lower local anesthetic concentration. In the setting of planned induction, this reduced concentration minimized the impact on labor progression while still providing sufficient analgesia.

## References

[1] Butwick AJ, Bentley J, Wong CA, Snowden JM, Sun E, Guo N (2018). United States state-level variation in the use of neuraxial analgesia during labor for pregnant women. JAMA Netw Open.

[2] Blondel B, Coulm B, Bonnet C, Goffinet F, Le Ray C (2017). Trends in perinatal health in metropolitan France from 1995 to 2016: results from the French National Perinatal Surveys. J Gynecol Obstet Hum Reprod.

[3] Maeda Y, Takahashi K, Yamamoto K, Tanimoto T, Kami M, Crump A (2019). Factors affecting the provision of analgesia during childbirth, Japan. Bull World Health Organ.

[4] Anim-Somuah M, Smyth RM, Cyna AM, Cuthbert A (2018). Epidural versus non-epidural or no analgesia for pain management in labour. Cochrane Database Syst Rev.

[5] Rojansky N, Tanos V, Reubinoff B, Shapira S, Weinstein D (1997). Effect of epidural analgesia on duration and outcome of induced labor. Int J Gynaecol Obstet.

[6] Studd JWW, Duignan ND, Selwyn CJ, Rowbotham CJF, Hughes A (1982). The effect of epidural analgesia on the progress and outcome of induced labour. J Obstet Gynaecol.

[7] Antonakou A, Papoutsis D (2016). The effect of epidural analgesia on the delivery outcome of induced labour: a retrospective case series. Obstet Gynecol Int.

[8] Callahan EC, Lee W, Aleshi P, George RB (2023). Modern labor epidural analgesia: implications for labor outcomes and maternal-fetal health. Am J Obstet Gynecol.

[9] Middleton P, Shepherd E, Morris J, Crowther CA, Gomersall JC (2020). Induction of labour at or beyond 37 weeks' gestation. Cochrane Database Syst Rev.

[10] Sargunam PN, Bak LL, Tan PC (2019). Induction of labor compared to expectant management in term nulliparas with a latent phase of labor of more than 8 hours: a randomized trial. BMC Pregnancy Childbirth.

[11] Swift EM, Gunnarsdottir J, Zoega H, Bjarnadottir RI, Steingrimsdottir T, Einarsdottir K (2022). Trends in labor induction indications: a 20-year population-based study. Acta Obstet Gynecol Scand.

[12] Glantz JC (2005). Elective induction vs. spontaneous labor associations and outcomes. J Reprod Med.

[13] (2025). American Society of Anesthesiologists. Statement on ASA Physical Status Classification System. https://www.asahq.org/standards-and-practice-parameters/statement-on-asa-physical-status-classification-system.

[14] Hurwitz EE, Simon M, Vinta SR, Zehm CF, Shabot SM, Minhajuddin A, Abouleish AE (2017). Adding examples to the ASA-physical status classification improves correct assignment to patients. Anesthesiology.

[15] Koyucu RG, Demirci N (2017). Effects of pushing techniques during the second stage of labor: a randomized controlled trial. Taiwan J Obstet Gynecol.

[16] Halpern SH, Abdallah FW (2010). Effect of labor analgesia on labor outcome. Curr Opin Anaesthesiol.

[17] Sun M, Chen Y, Sun L, Deng Y, Xu X, Zhang L, Xiong X (2025). Determination the ED90s of different concentrations of initial ropivacaine volume for labor analgesia with dural puncture epidural: a randomized sequential allocation study. Drug Des Devel Ther.

[18] Mao J, Chen Y, Sun L (2024). A randomized sequential allocation study on the optimum programmed intermittent epidural boluses interval time with different concentrations of ropivacaine combined with the dural puncture epidural technique for labor analgesia. Front Pharmacol.

[19] Hong JY, Jee YS, Jeong HJ, Song Y, Kil HK (2010). Effects of epidural fentanyl on speed and quality of block for emergency cesarean section in extending continuous epidural labor analgesia using ropivacaine and fentanyl. J Korean Med Sci.

[20] Liu H, Yao S, Rosinia F (2013). Low concentration lidocaine (0.5%) bolus epidurally can initiate fast-onset, effective and safe analgesia for early stage labor. Middle East J Anaesthesiol.

[21] Chen J, Chen S, Lv H, Lv P, Yu X, Huang S (2024). Using part of the initial analgesic dose as the epidural test dose did not delay the onset of labor analgesia: a randomized controlled clinical trial. BMC Pregnancy Childbirth.

[22] Zhu A, Pei L, Liu W, Cheng W, Zhang Y, Huang Y (2022). Neurologic complication due to crystallization after drug interaction between alkalized lidocaine and ropivacaine: a case report and in vitro study. Front Med (Lausanne).

[23] Liao JB, Buhimschi CS, Norwitz ER (2005). Normal labor: mechanism and duration. Obstet Gynecol Clin North Am.

[24] American Journal of Obstetrics and Gynecology (2014). Obstetric care consensus no. 1: safe prevention of the primary cesarean delivery. Obstet Gynecol.

